# Retraction Note: Psychometric properties of a short version of the Job Anxiety Scale

**DOI:** 10.1186/s12874-020-01166-w

**Published:** 2020-11-30

**Authors:** Bjarne Schmalbach, Andreas Kalkbrenner, Markus Bassler, Andreas Hinz, Katja Petrowski

**Affiliations:** 1 Department of Psychosomatic Medicine and Psychotherapy, University Medical Center of the Johannes Gutenberg University Mainz, Untere Zahlbacher Str. 8, 55131 Mainz, Germany; 2 Technische Universität Dresden, University Hospital Carl Gustav Carus, Clinic of Psychotherapy and Psychosomatic Medicine, Fetscherstraße 74, 01307 Dresden, Germany; 3 Hochschule Nordhausen, University of Applied Sciences, Weinberghof 4, 99734 Nordhausen, Germany; 4 Department of Medical Psychology and Medical Sociology, University of Leipzig, Philipp-Rosenthal-Str. 55, 04103 Leipzig, Germany; 5 University Medical Center of the Johannes Gutenberg University Mainz, Medical Psychology and Medical Sociology, Duesbergweg 6, 55131 Mainz, Germany

**Retraction to: BMC Med Res Methodol 20, 87 (2020)**

**https://doi.org/10.1186/s12874-020-00974-4**

The authors are retracting this article [1] because the Job Anxiety Scale has been modified without permission from the copyright holder.

All authors but one agree with this retraction. Markus Bassler has not responded to any correspondence from the editor about this retraction.
